# Molecular Epidemiology and Evolution of European Bat Lyssavirus 2

**DOI:** 10.3390/ijms19010156

**Published:** 2018-01-05

**Authors:** Lorraine M. McElhinney, Denise A. Marston, Emma L. Wise, Conrad M. Freuling, Hervé Bourhy, Reto Zanoni, Torfinn Moldal, Engbert A. Kooi, Antonie Neubauer-Juric, Tiina Nokireki, Thomas Müller, Anthony R. Fooks

**Affiliations:** 1 Wildlife Zoonoses and Vector Borne Disease Research Group, Animal and Plant Health Agency (APHA), Surrey KT15 3NB, UK; Denise.Marston@apha.gsi.gov.uk (D.A.M.); emma.wise@phe.gov.uk (E.L.W.); Tony.Fooks@apha.gsi.gov.uk (A.R.F.); 2 Institute of Global Health, University of Liverpool, Liverpool L69 3BX, UK; 3 Institute of Molecular Virology and Cell Biology, Friedrich Loeffler Institute, (FLI), 17493 Greifswald, Germany; Conrad.Freuling@fli.de (C.M.F.); Thomas.Mueller@fli.de (T.M.); 4 Institut Pasteur, Unit Lyssavirus Dynamics and Host Adaptation, 75015 Paris, France; hbourhy@pasteur.fr; 5 Institute of Virology and Immunology, University of Berne, 3012 Berne, Switzerland; reto.zanoni@vetsuisse.unibe.ch; 6 Norwegian Veterinary Institute, 0454 Oslo, Norway; torfinn.moldal@vetinst.no; 7 Wageningen Bioveterinary Research, 8221 RA Lelystad, The Netherlands; Bart.Kooi@wur.nl; 8 Bavarian Health and Food Safety Authority, 85764 Oberschleissheim, Germany; antonie.neubauer-juric@lgl.bayern.de; 9 Finnish Food Safety Authority Evira, 00790 Helsinki, Finland; tiina.nokireki@evira.fi

**Keywords:** EBLV-2, genome, Europe, evolution, epidemiology, *Myotis*, bat

## Abstract

Bat rabies cases in Europe are mainly attributed to two lyssaviruses, namely European Bat Lyssavirus 1 (EBLV-1) and European Bat Lyssavirus 2 (EBLV-2). Prior to the death of a bat worker in Finland in 1985, very few bat rabies cases were reported. Enhanced surveillance in the two subsequent years (1986–1987) identified 263 cases (more than a fifth of all reported cases to date). Between 1977 and 2016, 1183 cases of bat rabies were reported, with the vast majority (>97%) being attributed to EBLV-1. In contrast, there have been only 39 suspected cases of EBLV-2, of which 34 have been confirmed by virus typing and presently restricted to just two bat species; *Myotis daubentonii* and *Myotis dasycneme*. The limited number of EBLV-2 cases in Europe prompted the establishment of a network of European reference laboratories to collate all available viruses and data. Despite the relatively low number of EBLV-2 cases, a large amount of anomalous data has been published in the scientific literature, which we have here reviewed and clarified. In this review, 29 EBLV-2 full genome sequences have been analysed to further our understanding of the diversity and molecular evolution of EBLV-2 in Europe. Analysis of the 29 complete EBLV-2 genome sequences clearly corroborated geographical relationships with all EBLV-2 sequences clustering at the country level irrespective of the gene studied. Further geographical clustering was also observed at a local level. There are high levels of homogeneity within the EBLV-2 species with nucleotide identities ranging from 95.5–100% and amino acid identities between 98.7% and 100%, despite the widespread distribution of the isolates both geographically and chronologically. The mean substitution rate for EBLV-2 across the five concatenated genes was 1.65 × 10^−5^, and evolutionary clock analysis confirms the slow evolution of EBLV-2 both between and within countries in Europe. This is further supported by the first detailed EBLV-2 intra-roost genomic analysis whereby a relatively high sequence homogeneity was found across the genomes of three EBLV-2 isolates obtained several years apart (2007, 2008, and 2014) from *M. daubentonii* at the same site (Stokesay Castle, Shropshire, UK).

## 1. Introduction

There are currently 14 recognised species in the lyssavirus genus of the family *Rhabdoviridae* [1]. These species are comprised of the following viruses: rabies virus (RABV), Lagos bat virus (LBV), Mokola virus (MOKV), Duvenhage virus (DUVV), European bat lyssavirus 1 (EBLV-1), European bat lyssavirus 2 (EBLV-2), Australian bat lyssavirus (ABLV), Aravan virus (ARAV), Khujand virus (KHUV), Irkut virus (IRKV), West Caucasian bat virus (WCBV) Shimoni bat virus (SHIBV), Ikoma virus (IKOV), and Bokeloh bat lyssavirus (BBLV). A further two putative bat lyssaviruses await classification: Lleida lyssavirus (LLEBV) [2] and Gannoruwa bat lyssavirus (GBLV) [3]. With the exception of MOKV and IKOV, all lyssaviruses have been isolated from bats.

Cases of bat rabies occur globally, although different bat lyssavirus species are present in different regions [4,5]. Bat rabies cases in Europe are principally attributed to EBLV-1 and EBLV-2. However, three other lyssaviruses have been detected in European insectivorous bats, albeit in very few cases. WCBV was isolated in 2002 from a bent-winged bat (*Miniopterus schreibersii*) in the Caucasus Mountains and LLEBV from the same bat species in Spain in 2011 [2,6]. Antigenic and phylogenetic profiling of BBLV, isolated from *Myotis nattererii* in Germany and France, confirms the presence of a fifth lyssavirus in European bats [7,8,9].

The first record of a lyssavirus infected insectivorous bat in Europe was in Hamburg, Germany, in 1954 [10]. The reports described a biting incident from a bat that was caught and tested but not formally identified. Negri bodies were detected following mouse inoculation tests, but no tissue was retained for virus typing, so the bat and virus species involved are unknown. Prior to the death of a bat worker in Finland in 1985 [11], very few bat rabies cases were reported. Interestingly, 23% (263 cases) of all subsequent reported cases were identified through enhanced surveillance in the subsequent two years (1986 and 1987). With the advent of molecular PCR assays (polymerase chain reaction) and subsequent sequencing, the viruses were genetically characterised as two separate lyssavirus species, EBLV-1 and -2 [12].

Between 1977 and 2016, 1183 infected bats were reported in Europe with an average of 34 infected bats reported per year to Rabies Bulletin Europe in the last decade. More than 80% of the reported cases were identified in three countries the Netherlands (33%), Germany (27%), and Denmark (21%). Differences in surveillance systems, bat ecology, public awareness, and available resources may account for the heterogeneous spatial pattern of confirmed bat rabies cases [13]. However, the apparent host specificity may also limit the prevalence of bat lyssaviruses when considering the wide distribution, high frequency, and sympatric habitation of some European insectivorous bat species. Whilst lyssaviruses have the ability to cause clinical disease in bats, they can also circulate within healthy bat populations detectable only via active surveillance screening [14,15].

Cross species transmission (CST) of bat lyssaviruses to other mammals has been documented but is extremely rare [16]. Two CST events involving EBLV-2 from bats to humans have been reported, in Finland in 1985 and in the UK in 2002. Unlike for EBLV-1, EBLV-2 spillover into other wildlife or domestic species has not been documented and no other lyssavirus has yet been isolated from *M. Daubentonii* or *M. dasycneme*.

Evolutionary analyses, sub-clinical infections and restricted virus–host relationships suggest that all lyssaviruses, including RABV, most likely originated in bats [16,17,18]. Previous attempts to study the phylogeography, molecular epidemiology, and evolutionary rates for EBLV-2 have been restricted to only a small number of virus isolates or partial genomic sequences [19,20,21]. The historic sharing of viruses between European rabies laboratories for confirmation and research was not standardised. This is particularly evident for the EBLV-2 viruses due to the small number involved. However, differences in virus handling (multiple passages in vivo and in vitro), various sequencing approaches (direct sequencing from amplicons, sequencing from cloned products, and deep sequencing), and improvements in the proof reading fidelity of molecular enzymes have resulted in anomalous published sequences being submitted for the same isolate [20]. To address this, a network of rabies reference laboratories involved in EBLV-2 surveillance and diagnosis was established to review, collate, and analyse all available EBLV-2 submissions. The aim of this review was to collate and analyse a comprehensive and accurate genomic sequence dataset for EBLV-2 providing researchers with both epidemiological and genetic data for every confirmed case. Furthermore, whole genome sequences from the same site were analysed to understand the diversity of viruses within the same maternity roost.

## 2. Results and Discussion

### 2.1. Review of Reported Cases

**Figure 1:**
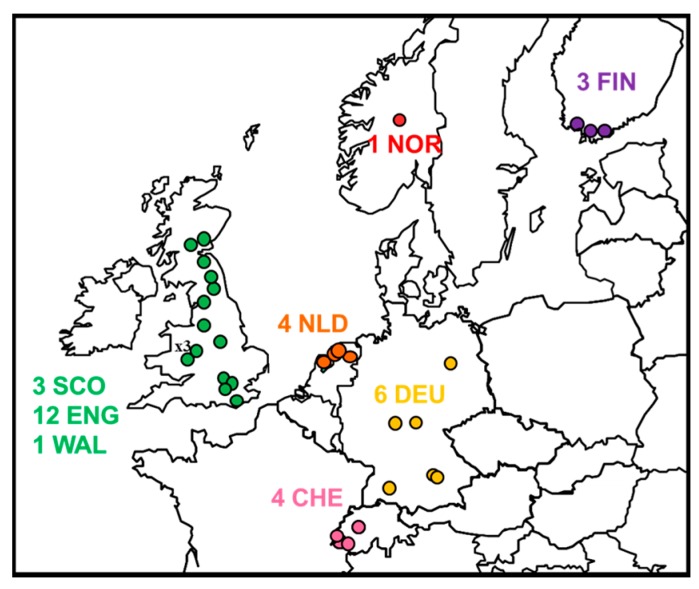
Distribution of 34 confirmed cases of EBLV-2 (Table 1) in Europe between 1985 and 2017 (WHO Rabies Bulletin Europe).

Data from all potential cases of EBLV-2 were collated. The distribution and submission details are summarised in Figure 1 and Table 1 and include confirmed cases of EBLV-2.

EBLV-2 was first isolated in 1985 from a Swiss bat biologist who had been working with bats in Finland, Switzerland, and Malaysia. In 1986, EBLV-2 was isolated in Switzerland from *M. daubentonii* (Daubenton’s bats). Between 1985 and 2017, a total of 39 cases are suspected to have been associated with EBLV-2, of which 34 have been confirmed (Table 1). Full genome sequences have been previously published or obtained in this study for 29 EBLV-2. In addition, EBLV-2 RNA has been detected by RT-PCR in oro-pharyngeal swabs of two apparently healthy *M. daubentonii* bats during active surveillance (Scotland and Switzerland), yielding partial N gene sequences (Table 1). Unfortunately, sequence data obtained for three outstanding EBLV-2 cases (UK RV3385, Germany F860/LGL and 2Finland2016) were obtained too late to be included in this analysis. Assuming that rabies in *M. daubentonii* and *M. dasycneme* (Pond bats) is restricted to EBLV-2 infection, there were three further probable EBLV-2 cases in Denmark from single *M. dasycneme* (1986, 1987) and *M. daubentonii* (1986) bats [22]. In Germany, bat rabies was diagnosed in two *M. daubentonii* in 1986 [23]. However, no material is available for confirmation of an EBLV-2 infection from these probable cases so they remain unconfirmed. The total number of confirmed (typed) EBLV-2 cases is relatively small compared to EBLV-1 isolated from *Eptesicus* bat species. This discrepancy could be due to less effective surveillance in non-synanthropic bat species like Daubenton’s bats compared to *Eptesicus* bats or the low transmission rates of EBLV-2 among conspecifics as indicated by experimental studies in Daubenton’s bats [24].

Fourteen cases of EBLV-2 in *M. daubentonii* were reported through passive surveillance in the UK between 1996 and 2017 and a single human case in a male in Scotland in 2002 [29]. Unfortunately, sequence data from the latest UK case in September 2017 (Table 1, Derbyshire, England) was obtained too late to be included in the phylogenetic analysis in this review. However, preliminary sequence analysis (N1356) suggests a closer identity (99.6%) to the 1996 EBLV-2 RV628 and 2006 EBLV-2 RV2159 cases (Table 1, Sussex and Oxfordshire, respectively). In addition, EBLV-2 viral RNA was detected in a single oro-pharyngeal swab specimen collected from a healthy Daubenton’s bat sampled as part of an active surveillance programme in Scotland in 2008 [20].

The discovery of EBLV-2 in *M. daubentonii* in Finland (2009) provided evidence for the possible origin of human infection in 1985 [40]. Prior to this latter case, surveillance had failed to detect EBLV-2 in Finland despite intensive efforts in 1986 by active surveillance [45] and continued passive surveillance since (albeit for low submissions averaging 10/year). Subsequent active surveillance has failed to detect viral RNA in the oro-pharyngeal swabs of healthy Daubenton’s bats [46]. A second case of EBLV-2 in *M. daubentonii* has more recently been reported in Inkoo, Southern Finland (October 2016) but genome sequence case was not available for this review [44]. However, partial N gene data (N253, accession number MF326269) confirms a closer sequence identity (97.6–98%) to the previous 2 Finland EBLV-2 cases than to any other EBLV-2 (91.7–95.7%).

In Germany, six rabies cases in Daubenton’s bats have been typed as EBLV-2 [33,34,35,41], and five genome sequences were available to be included in this study. Notably, only one case was detected in the frame of routine rabies surveillance, whilst the other five isolates were only found during enhanced surveillance programmes targeting all dead found bats [34]. Genomic sequence data for the outstanding German case (Table 1, Bavaria 2013 860/LGL) were obtained too late to be included in this study, but preliminary sequence analysis (N1336) confirms the closest sequence identity to the 2012 Bavarian German EBLV-2 F500/LGL (99.8%). 

The Netherlands is the only country where EBLV-2 was detected in *M. dasycneme* bats [47], of which sequences are available for four isolates (Table 1). Interestingly, passive surveillance has not yielded any further EBLV-2 cases since 1993.

In Switzerland, passive surveillance between 1976 and 2009 revealed three cases of EBLV-2, all in *M. daubentonii* and a single PCR positive oro-pharyngeal swab sample via active surveillance [26]. The three Swiss EBLV-2 isolates identified through passive surveillance have previously been analysed [19,20,26,48], but discrepancies in the sequence obtained for the three isolates were noted between different studies. In addition, it is unclear if the partial N gene sequence published as U89479, which shows a number of mismatches compared to all other Swiss EBLV-2 sequences, represents an additional Swiss virus or is a result of erroneous sequencing. In order to solve these issues, original virus isolates for the Swiss EBLV-2 held at APHA and University of Berne were subject to NGS and the correct genome sequences were obtained in this study (Table 1).

The first case of EBLV-2 in Norway was reported in a Daubenton’s bat in 2015. The bat was found by a member of the public in Oppland, in the southern part of Norway [42].

### 2.2. Phylogenetic Analysis

**Figure 2:**
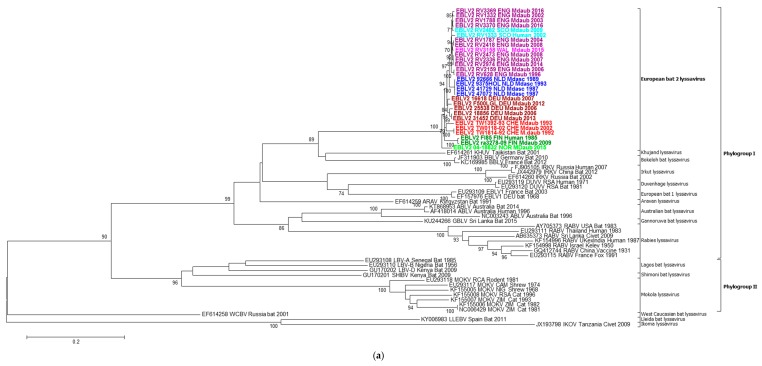

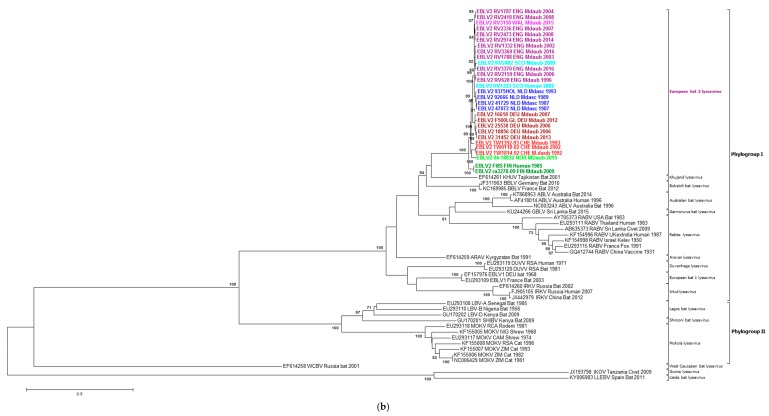
Molecular phylogenetic analysis of (**a**) nucleoprotein sequences (1353 nucleotides) and (**b**) glycoprotein sequences (1569 nucleotides) from 29 EBLV-2 cases and 36 non EBLV-2 lyssavirus sequences (*n* = 65). Sequences were analysed by the Maximum Likelihood method. Bootstrap values > 70% are shown for each branch and analysis was performed using the GTR + G + I model in MEGA6 with 1000 bootstrap samples. The EBLV-2 sequences are coloured depending upon sampling location (see 3 letter codes within case names) (**a**) N1353; (**b**) G1569.

**Figure 3:**
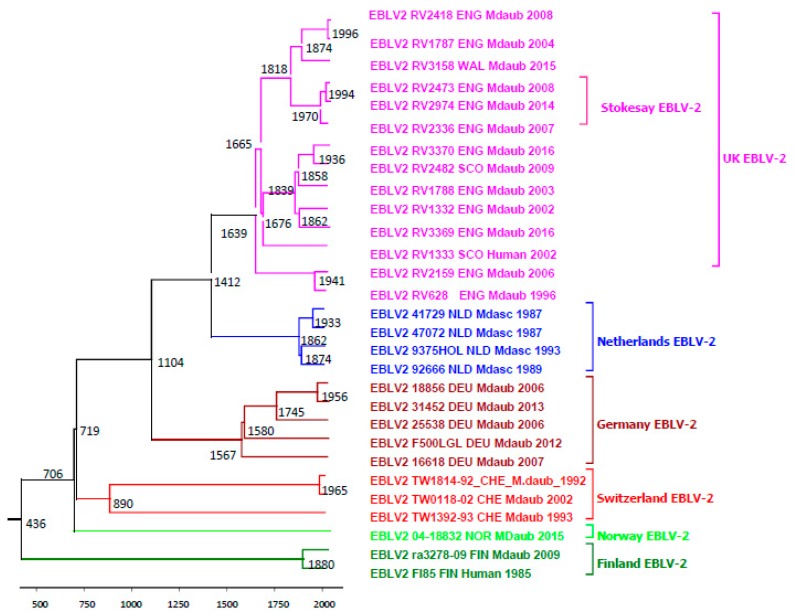
Bayesian maximum clade credibility (MCC) tree of 29 EBLV-2 concatenated gene sequences (10818 nucleotide sites comprising the nucleoprotein, matrix, phosphoprotein, glycoprotein, and polymerase genes) with branch lengths scaled in time by enforcing a relaxed molecular clock. The EBLV-2 strains are coloured to indicate the location of sampling. Mean substitution rate of the dataset was 1.7 × 10^−5^.

The phylogenetic analysis confirmed the close relationship of EBLV-2 sequences to the Phylogroup I lyssaviruses, in particular KHUV and BBLV, irrespective of the gene studied (Figure 2a,b, maximum likelihood trees for other genes not shown). Where available, a number of representative lineages for the lyssaviruses were included. The genetic diversity observed for the other lyssavirus species is in contrast to the highly homogeneous sequence identities observed for EBLV-2. However, despite this homogeneity, the EBLV-2 sequences are resolved geographically for all analyses, with clear clustering at the country level. Even within country, resolution can be observed when sufficient numbers allow. The Scotland and Northern England EBLV-2 isolates can be seen to cluster separately to the Southern England and Wales EBLV-2 isolates, although bootstrap support is not consistent (Figure 2 and Figure 3). The human EBLV case (RV1333) in Scotland in 2002, resolved closest to the Northern England and Scotland cases. In the partial N400 analysis (not shown), human UK EBLV-2 was most similar to the partial sequence obtained from a Daubenton’s saliva sample during active surveillance in Scotland [20]. Although originating from disparate regions in the south and north of Germany, the German EBLV-2 viruses cluster together and are most closely associated with the 2002 Swiss isolate (TW0118/02) (Figure 2). All of the Dutch EBLV-2 sequences cluster separately from the other sequences possibly reflecting either geographical and/or host species relationships. The latter may be likely as the EBLV-2 isolates from the Netherlands are the only ones sampled from *M. dasycneme*. The Norwegian EBLV-2 is distinct but is most closely related to the Swiss EBLV-2.

The two available EBLV-2 genome sequences from Finland are clearly distinct from the other EBLV-2 studied and have previously been proposed as a separate lineage, although depending upon the gene studied, the bootstrap support is not always consistent. However, the relatively low number of available EBLV-2 sequences makes it difficult to confidently further divide into monophyletic sub lineages. Although, the subtypes 2a and 2b of EBLV-2 were proposed in earlier studies [48], it has more recently been reported that the highest nucleotide sequence divergence was between two isolates (AY863408—Switzerland isolate and AY863406—Finland human case), previously assigned as EBLV-2b [49]. The two EBLV-2 sequences from Finland [21] and the erroneous sequence U89479 consistently formed a well-supported sub lineage (EBLV-2b?) irrespective of the model used (bootstrap support 100% for Neighbour Joining (*p*-values) and 99% for Maximum Likelihood (GTR) models). In contrast, the 1992 & 1993 Swiss EBLV-2 sequences were not consistently associated with either sub-lineage when individual sequences were removed or additional lyssavirus sequences added.

Intra-genotypic comparisons were reported previously for the five viral proteins of EBLV-2 [49] and demonstrated the limited genetic diversity of these lyssaviruses compared to RABV at both the nucleotides and amino acid levels. In these analyses, using concatenated gene sequences, the similarity of the EBLV-2 ranged from 95.5–100% for nucleotides and from 98.7–100% for amino acids.

With respect to the anomalous sequence U89479, no evidence has been found to support it as an additional EBLV-2 sequence, hence we conclude it is erroneous and recommend it is not used in future EBLV-2 analyses.

### 2.3. Intra-Roost Variation

The three EBLV2 isolates from Daubenton’s bats found at Stokesay castle (Shropshire, UK) were studied in greater detail as they represented the only cluster of EBLV-2 with three separate isolations at the same site in 2007, 2008, and 2014. The percentage of viral reads assembled from the total reads varied between isolates, ranging from 0.98% (RV2473) to 2.08% (RV2336). Depth of coverage (average and maximum) was variable and correlated with the number of viral reads assembled (Table S1).

**Figure 4:**
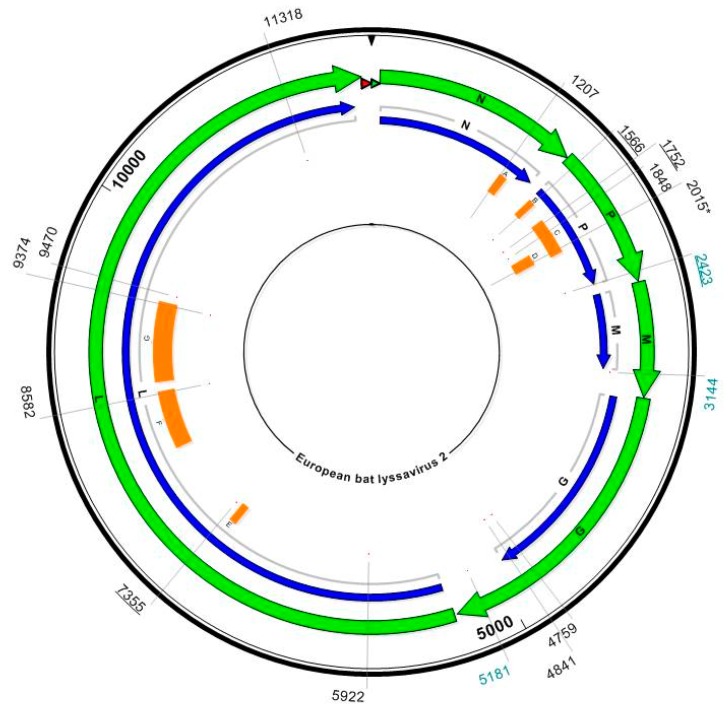
A circular map of the EBLV2 genome. Numbers indicate genomic positions at which heterogeneity is seen between the three genomes from Stokesay Castle. Black positions are located within coding regions, teal positions are in non-coding regions. Position 2015 (asterisk) has a base change corresponding to an amino acid substitution. Orange blocks indicate known domains within each protein where heterogeneity between the three genomes is observed. Underlined positions indicate those at which >1% heterogeneity is seen within the host. Domains key: A = T-cell epitopes; B = L binding region; C = soluble N binding region; D = LC8 protein binding region; E = Domain III; F = Domain IV; G = Domain V.

The three genomes were aligned and found to be 99.9% identical to one another. Positions at which single nucleotide polymorphisms (SNPs) occurred were identified and investigated to determine whether they denote a change in amino acid within a region of the genome coding for a viral protein (Figure 4). Surprisingly, only one such SNP was identified within the P gene at Position 2015, despite up to a 7-year interval between isolations. RV2336 and RV2974 code for lysine at this position, whereas the change in base from A to G in RV2473 results in the translation of arginine instead (Table S2). This SNP is located within the LC8 domain (cytoplasmic dynein light chain) at residue 164. The LC8 domain has been previously identified as a region at which the P protein interacts with LC8, a cytoplasmic protein associated with axonal transport of RABV within neurons [49,50], but the SNP is not within the key residues of 145–149, the motif to which LC8 binds. In addition, the substitution is considered conservative due to the similar biochemical properties of the amino acids.

To investigate heterogeneity of the 3 Stokesay Castle isolates across the length of each genome, each position in the genome was analysed to determine the number of reads showing each Base A, T, C, or G. The proportion of reads showing each base was calculated and plotted to give a graphical representation of heterogeneity across the genome for each of the three EBLV-2 isolates. Data was filtered to remove positions that showed less than 100 reads, and less than or equal to 1% heterogeneity. Across the length of the genome, RV2336 has 23 positions at which heterogeneity equals or exceeds 1%, RV2473 has 257 positions and RV2974 has 212. The similar level of heterogeneity between RV2473 and RV2974 gives us confidence that this data is not merely a reflection of the varying depth of coverage between the genomes; RV2473 has the lowest average depth of coverage (245 reads) whilst RV2974 has the highest (970 reads). There are three positions at which heterogeneity equals or exceeds 2% in RV2336, 50 positions in RV2473, and 86 positions in RV2974. Additional analysis and figures can be found in the Supplementary data, Supplementary Tables S1 and S2 and Supplementary Figure S1.

### 2.4. Evolutionary Analysis of EBLV-2

Evolutionary analysis was undertaken using an alignment of concatenated genes for the 29 EBLV-2 sequences. The analysis supports the previously observed relatively low evolutionary rates for EBLV-2 compared to other RNA viruses [19,21]. The ancestral root of the available EBLV-2 concatenated dataset was likely to have diverged as far back as 436AD, but the 95% HPD intervals are wide (−9553 to 1655). The recent ancestors of the geographical clusters of EBLV-2 are estimated as follows (Figure 3); Finland in 1880 (range 1133 to1969), Norway in 706 (single isolate −7535 to 1664); Switzerland in 890 (−6280 to 1734), Germany in 1567 (−1326 to 1910); Netherlands in 1862 (1042 to 1961); UK in 1639 (−727 to 1923). The Stokesay EBLV-2 ancestors are estimated to have existed much more recently in 1970 (1725 to 2003). The long TMRCAs observed (Figure 3) is suggestive of minimal and discrete evolution at the local level (Figure 3). Migration of infected bats, whilst possible, does not appear to be supported in our dataset. Mean rate of evolution for the concatenated genes for the EBLV-2 dataset (*n* = 29) was 1.7 × 10^−5^ (4.65 × 10^−8^, 3.63 × 10^−5^).

## 3. Materials and Methods

Firstly, a literature review was undertaken of all reported cases of EBLV-2 between 1985 and 2017. As well as peer-reviewed literature and published sequences on GenBank, other sources included Rabies Bulletin Europe Reports, ProMed reports, and reference laboratory archives. When available and where gaps existed, original brain material was sought to clarify anomalies in published data and obtain complete genome sequences (Table 1).

### 3.1. Next Generation Sequencing (NGS)

Total RNA (from original brain material) was depleted of host genomic DNA (gDNA) and ribosomal RNA (rRNA) following methods described previously [51,52]. Briefly, gDNA was depleted using the on-column DNase digestion protocol in RNeasy plus mini kit (Qiagen, Manchester, UK) following manufacturer’s instructions, eluting in 30 µL in molecular grade water. Subsequently, rRNA was depleted, using Terminator 5′-phosphate-dependent exonuclease (CamBio, Cambridge, UK). Briefly, 30 µL of gDNA depleted RNA was mixed with 3 µL of Buffer A, 0.5 µL of RNAsin Ribonuclease inhibitor (20–40 U/µL) (Promega, Southampton, UK), and incubated at 30 °C for 60 min. The depleted RNA was purified to remove the enzyme using the RNeasy plus mini kit as above, without the DNase digestion, eluting in 30 µL of molecular grade water. ds cDNA (double-stranded copy DNA) was synthesised using random hexamers and a cDNA synthesis kit (Roche, Burgess Hill, UK) following manufacturer’s instructions. The resulting ds-cDNA was purified using AMPure XP magnetic beads (Beckman Coulter, High Wycombe, UK), quantified using Quantifluor (Promega) and approximately 1 ng of each sample was used in a “tagmentation” reaction mix using a Nextera XT DNA sample preparation kit (Illumina, San Diago, CA, USA) following the manufacturer’s instructions—without the bead normalization step. DNA libraries were quantified using Quantifluor (Promega). Individual libraries were pooled (approximately 8–12 samples per MiSeq run) and normalized to equimolar concentrations. Libraries were sequenced as 2 × 150 bp paired-end reads on an Illumina MiSeq platform (Illumina).

### 3.2. Mapping to Reference Sequence to Obtain Complete Genome Sequences

Reads were mapped using the Burrow–Wheeler Aligner (BWA version 0.7.5a–r405) and were visualized in Tablet [53]. A modified SAMtools/vcfutils [54] script was used to generate an intermediate consensus sequence in which any indels and SNPs relative to the original reference sequence were appropriately called. The intermediate consensus sequence was used as the reference for four subsequent iterations of mapping and consensus calling. Sequencing resulted in 100% coverage of the five genes for all samples. Consensus sequences were submitted to Genbank (accession numbers provided in Table 1).

### 3.3. Evolutionary Analysis

Sequences generated in this study were combined with published EBLV-2 sequences and aligned using ClustalX2 (version 1.2, www.clustal.org/clustal2). Nucleotide and amino acid identities were obtained using MegAlign, v11.1.0 (Lasergene DNASTAR. Madison, WI, USA).

A phylogenetic analysis of all 29 EBLV-2 concatenated coding gene sequences (Accession numbers provided in Table 1) was undertaken using Bayesian Markov Chain Monte Carlo simulation in the BEAST package v1.8.0 [55]. A GTR nucleotide substitution model with rate variation and a proportion of invariant sites (Gamma) were determined to best fit the data using Akaike Information Criterion (AIC) in MEGA 6.0 [56]. A relaxed molecular clock model with constant population prior were used in Markov Chain Monte Carlo (MCMC) simulation for 100,000,000 iterations, sampling every 10,000 states to give effective sample sizes of over 200. Molecular clock and population coalescent models were compared using a modified AIC (AICM) in Tracer v1.5, as described previously [57], and maximum clade credibility trees were annotated using TreeAnnotator (v1.8.4) after 10% of trees (burn-in) were discarded [55]. The maximum clade credibility (MCC) trees were then visualised using Fig Tree (v1.4.3, http://tree.bio.ed.ac.uk/software/figtree/). Maximum likelihood phylogenetic reconstructions were also undertaken on the EBLV-2 dataset (*n* = 29) in MEGA 6.0 [56] for each individual gene and for partial N gene sequences (400 nucleotides).

## 4. Conclusions

This review represents the most comprehensive and accurate genomic analysis of EBLV-2 to date and includes the first detailed intra-roost genetic analysis of EBLV-2 isolated from the same site between 2007 and 2014.

The chiropteran host range of confirmed EBLV-2 appears to be restricted to two bat species *M. Daubentonii* (*n* = 28) and *M. dasycneme* (*n* = 5) (Table 1)*.* The distribution of *M. dasycneme* is confined to Central and Eastern Europe, whereas *M. daubentonii* is widely distributed throughout Europe [58]. *M. dasycneme* is considered to be absent in the UK. However, given the relationship between bat species and their lyssaviruses [4], it remains unclear as to whether *M. dasycneme* is a true reservoir for EBLV-2 or whether these cases represent spill over infections. The genetic structure of *M. daubentonii* in Western Europe is relatively homogenous and data suggests migratory movements between the UK and the mainland Europe by Daubenton’s bats [59]. Many factors affecting the behaviour of Daubenton’s bats, may allow EBLV-2 to become established and rapidly spread throughout the population. This may account for the greater detection of EBLV-2, albeit at low levels, throughout the UK [60]. However, despite the long distance migratory behaviour of Daubenton’s bats [61] and the extended distribution of EBLV-2 across Europe, there is a striking spatial clustering of EBLV-2 sequences, irrespective of the genomic region analysed.

Within EBLV-2 there is a high level of sequence homogeneity and low evolutionary rate compared to other lyssaviruses. The intra-roost analysis of the three Stokesay EBLV-2 genomes sampled within a 7-year period demonstrates extremely few SNPs and insignificant levels of heterogeneity in the viral reads. However, partial sequence analysis would have been limited in differentiating the isolates, thus confirming the importance of generating full genome/concatenated gene data to improve resolution. The low mean substitution rate of 1.7 × 10^−5^ of the EBLV-2 concatenated gene dataset is similar to the 4.19 × 10^−5^ rate recently determined for EBLV-1 [62] but contrasts starkly with rates published for similar concatenated gene or complete genome datasets for RABV, e.g., 3.10 × 10^−4^ for Turkey canine RABV dataset [63], a comprehensive canine RABV dataset 2.44 × 10^−4^ [64] and a fox RABV dataset from Greenland 2.5 × 10^−4^ [65]. The long TMRCAs and low substitution rate suggests limited and localised evolution has occurred over very long periods of time. The distribution and evolutionary analysis suggest an east–west axis possibly in an east (Finland) to west (UK) direction (Figure 1 and Figure 3).

In recent years, multiple novel lyssaviruses have been isolated from bats. Passive bat surveillance, complemented by active surveillance where appropriate, will facilitate the further isolation of known and novel bat lyssaviruses. To fully assess the diversity and evolution of bat lyssaviruses in Europe, all isolates from bat rabies cases should be archived and further characterized. Molecular typing of wildlife rabies cases will facilitate the early detection of CST events which could give rise to the adaption and maintenance of bat lyssaviruses in non-flying mammals. As confirmed in this study, NGS offers unique insights into viral evolution. For a virus with relatively few cases, it is prudent to obtain full genome sequence and study the possible causes for such limited viral evolution and restricted host range [16].

## Figures and Tables

**Table 1:** Confirmed cases of EBLV-2 in Europe 1985–2017 and sequences used to generate phylogenetic trees.

Year	Original Reference	APHA Virus Ref.	PI Virus Ref.	Location	Country	Species	N400 Data Accession No.	Genome Accession No.	Case or Sequence Ref.
1985	FINMAN	RV8	9007FIN	Helsinki	Finland	Human	AY062091	JX129233, KY688151	[11]
1987	41729	RV30	9018HOL	Wommels	Netherlands	*M.dasyc*	EU293114	EU293114	[19]
1987	47072	RV29	9482HOL	Tjerkwerd	Netherlands	*M.dasyc*	U89480	KY688145	[25]
1989	92666	RV228	94112HOL	Andijk	Netherlands	*M.dasyc*	AY062089	KY688146	[19]
1992	TW1814/92	RV594, RV2478	-	Plaffeien	Switzerland	*M.daub.*	AY212117	KY688133	[26]
1993	-	-	9375HOL	Roden	Netherlands	*M.dasyc*	AY863404	KY688152	[19]
1993	TW1392/93	RV621, RV2479	9337SWI	Versoix	Switzerland	*M.daub*	AY212118	KY688140	[19]
1996	18/96	RV628	EBL2GB	Sussex	England	*M.daub*	U89478	KY688136	[27]
2002	105/02	RV1332	-	Lancashire	England	*M.daub*	AY212120	KY688150	[28]
2002	Human case	RV1333	-	Angus	Scotland	Human	EF157977	EF157977	[29]
2002	TW0118/02	RV2480	02053SWI	Geneva	Switzerland	*M.daub*	AY863408	KY688132	[19]
2004	603/04	RV1787	-	Surrey	England	*M.daub*	JQ796807	KY688142	[30]
2003	696/04	RV1788	-	Lancashire	England	*M.daub*	JQ796808	KF155004	[31]
2006	672/06	RV2159	-	Oxfordshire	England	*M.daub*	JQ796809	KY688144	[32]
2006	18856	RV2506	-	Magdeburg	Germany	*M.daub*	JQ796805	KY688135	[33]
2006	25538	-	-	Thuringia	Germany	*M.daub*	KF826115	KY688137	[34]
2007	16618	RV2505	-	Bad Buchau	Germany	*M.daub*	GU227648	KY688138	[35]
2007	762/07	RV2336	-	Shropshire	England	*M.daub*	JQ796810	KY688139	[36]
2008	166/08	RV2418	-	Surrey	England	*M.daub*	JQ796811	KY688141	[37]
2008	1218/08	RV2473	-	Shropshire	England	*M.daub*	JQ796812	KY688147	[38]
2009	882/09	RV2482	-	West Lothian	Scotland	*M.daub*	JQ796806	KY688143	[39]
2009	ra3278/09	-	-	Nr Turku	Finland	*M.daub*	GU002399	JX129232	[40]
2012	F500/LGL	-	-	Ingolstadt, Bavaria	Germany	*M.daub*	-	KY688149	[34,41]
2013	31452	-	-	Gießen, Hesse	Germany	*M.daub*	KF826149	KY688134	[34]
2013	F860/LGL	-	-	Ingolstadt, Bavaria	Germany	*M.daub*	-	Not available	This study
2014	281/14	RV2974	-	Shropshire	England	*M.daub*	-	KY688148	This study
2015	421/15	RV3158	-	Newtown, Powys	Wales	*M.daub*	-	KY688153	This study
2015	2015-04-18832	-	-	Oppland	Norway	*M.daub*	-	KY688154	[42]
2016	307/16	RV3369	-	North Yorkshire	England	*M.daub*	-	KY688156	This study
2016	432/16	RV3370	-	Northumberland	England	*M.daub*	-	KY688155	[43]
2016	2Finland2016	-	-	Inkoo, Uusimaa	Finland	*M.daub*	MF326269	Not available	[44]
2017	591/17	RV3385	-	Derbyshire	England	*M.daub*	-	Not available	This study
**Active Surveillance Detection of Viral RNA in Oro-Pharyngeal Swab Only:**									
2008	80063	M08/09	-	Perthshire	Scotland	*M.daub*	JQ796804	Not available	[20]
2009	70	-	-	Genthod	Switzerland	*M.daub*	HM067110	Not available	[26]

Key—not known. *M.daub—Myotis daubentonii*; *M.dasyc—Myotis dasycneme*.

## Supplementary Materials

Supplementary materials can be found at www.mdpi.com/1422-0067/19/1/156/s1.
