# Supplementary material for: Molecular Epidemiology and Evolution of European Bat Lyssavirus 2
Source: Int J Mol Sci. 2018 Jan 5;19(1):156. doi: 10.3390/ijms19010156 (PMC5796105; doi:10.3390/ijms19010156)
Supplement: Supplementary file 1 [file ijms-19-00156-s001.pdf]

Supplementary Materials: Molecular Epidemiology and Evolution of European Bat Lyssavirus 2

Lorraine M McElhinney *, Denise A Marston, Emma L Wise, Conrad M Freuling, Hervé Bourhy, Reto Zanoni, Torfinn Moldal, Engbert A Kooi, Antonie Neubauer-Juric, Tiina Nokireki, Thomas Müller, Anthony R Fooks

Base substitutions were identified at three other positions, however these are within non-coding regions of the genome, therefore do not affect the viral proteins. This is not to say that mutations in non-coding regions are unimportant, as they may play a part in areas such as viral replication or structure. Minor differences in genome sequence exist between the three isolates from Stokesay castle. To investigate these differences further, we looked at the raw sequence reads at the positions where SNPs were shown, to identify whether or not viral variants were observed that differed from the consensus sequence at each given position (provided as supplementary data).

In the 2007 isolate (RV2336), at the positions at which SNPs are seen, no variants exceed 1% of the total viral reads, however a number of variants are seen, four of which result in an amino acid change, all of which are non-conservative changes (Table S1). At position 2423, within the P-M intergenic regions, the 2008 isolate (RV2473) shows a variant (T) that occurs in 1.01% of the total viral reads. None of the RV2473 variants shown result in an amino acid change. Three positions within the 2014 isolate (RV2974) show variants that exceed 1% of the total viral reads; 1.01% of reads at position 1566 (P gene) are A, 2.26% of reads at position 1752 (P gene) are A, and 1.05% of reads at position 7355 (L gene) are T (Table S2). Six variants observed result in amino acid changes; three of which are conservative, two of which are non-conservation and one of which results in a stop codon (position 9374).

We further investigated whether the variants seen at >1% of the total reads were observable at lower proportions previously, or if they became fixed over time. Position 1566 (P gene) is 99.7% C with a minority population of A in 2007. This changes dramatically by 2008, in which 100% of the reads are T at this position. In 2014, T remains the major variant, with two minor variants of A (>1%) and C (0.09%). Position 1752 (P gene) is 99.7% A with a minority population of G in 2007. In 2008 the minority population has disappeared and all reads show an A at this position. By 2014, the majority population has changed and 97.1% of the reads show a G, with a smaller remaining population of A (2.26%) and a minority population of T (0.6%). Position 2423 (P-M intergenic region) shows no variation in 2007; 100% of reads are C. In 2008, a minority T variant appears (1.05%), which has become fixed at this position by 2014 (99.84%). No C remains in 2014, however 0.16% of reads show A. Position 7355 (L gene) is 99.82% T in 2007, with a minority population of A. In 2008, 100% of reads have a C at this position, and by 2014 the majority population of C remains, with two minor variants of T (1.05%) and A (0.45%).

Table S1. Viral variants identified at positions with SNPs. Numbers indicate genomic positions within the three EBLV-2 genomes from Stokesay Castle. For each position the total number of reads is given, as well as the consensus base and whether variants from the consensus are seen. Where variants exist, the variant base is given as well as the percentage of viral reads that show this base. Variants that exceed 1% of the total viral reads are highlighted in yellow. * indicates an amino acid substitution. - indicates no variants at this position. Text in blue highlights the only SNP denoting an amino acid substitution within the consensus sequences.

		Total number of reads			Consensus base			Variant base(s) [% of viral reads with this variant]		
Genomic position	Genomic region	2007	2008	2014	2007	2008	2014	2007	2008	2014
1207	N gene	338	144	734	A	G	G	-	-	A 0.68%<br>T 0.51%
1566	P gene	236	136	710	C	T	T	A 0.30%	-	**A 1.01%**<br>C 0.09%
1752	P gene	297	139	593	A	A	G	G 0.27%	-	**A 2.26%**<br>*T 0.60%
1848	P gene	306	124	527	G	A	A	*T 0.26%	-	*T 0.14%
2015	P gene	259	152	580	A	G	A	-	-	-
2423	P-M	246	153	430	C	C	T	-	**T 1.05%**	A 0.16%
3144	M-G	433	149	600	T	C	C	-	-	A [0.22%]
4759	G gene	476	133	381	C	T	T	T 0.23%<br>A 0.18%	C 0.60%	A 0.34%
4814	G gene	534	154	449	C	C	T	*A 0.11%	T 0.54%	*G 0.16%
5181	G-L	382	121	343	C	T	T	-	-	A 0.26%
5922	L gene	487	162	425	C	T	T	-	-	*A 0.30%<br>*G 0.15%
7355	L gene	368	144	449	T	C	C	G 0.18%	-	**T 1.05%**<br>**A 0.45%**
8582	L gene	546	183	757	A	A	G	*C 0.11%	-	A 0.61%<br>T 0.43%
9374	L gene	476	204	881	T	C	C	*G 0.13%	-	T 0.75%<br>*A 0.69%
9470	L gene	498	228	821	A	G	G	C 0.12%<br>G 0.12%	-	T 0.35%<br>A 0.21%
11318	L gene	427	436	920	T	C	T	C 0.15%	T 0.31%	A 0.13%<br>C 0.06%

Table S2. SNPs identified between the three EBLV2 genomes from Stokesay Castle. Numbers indicate genomic positions within the three genomes. For each position, information on genomic region, domain within that region, position within amino acid and whether the SNP results in a substitution of a coding amino acid. Domain information from Marston et al. 2007. K = Lysine, R = Arginine. Colour highlighting indicates pattern of change in base at this position; red = different, same, same; green = same, same, different; blue = same, different, same.

			Base				
Genomic Region	Domain	Position	2007	2008	2014	Base Position within Amino Acid	Amino Acid Change in Coding Region?
N gene	T cell epitope	1207	A	G	G	3rd	No
P gene	L binding region	1566	C	T	T	3rd	No
P gene	N° binding region	1752	A	A	G	3rd	No
P gene	N° binding region	1848	G	A	A	3rd	No
P gene	LC8 binding region	2015	A	G	A	2nd	K-R-K
P-M	None	2423	C	C	T	n/a	No
M-G	None	3144	T	C	C	n/a	No
G gene	None	4759	C	T	T	3rd	No
G gene	None	4814	C	C	T	3rd	No
G-L	None	5181	C	T	T	n/a	No
L gene	None	5922	C	T	T	1st	No
L gene	L domain III	7355	T	C	C	3rd	No
L gene	L domain IV	8582	A	A	G	3rd	No
L gene	L domain V	9374	T	C	C	3rd	No
L gene	None	9470	A	G	G	3rd	No
L gene	None	11318	T	C	T	3rd	No

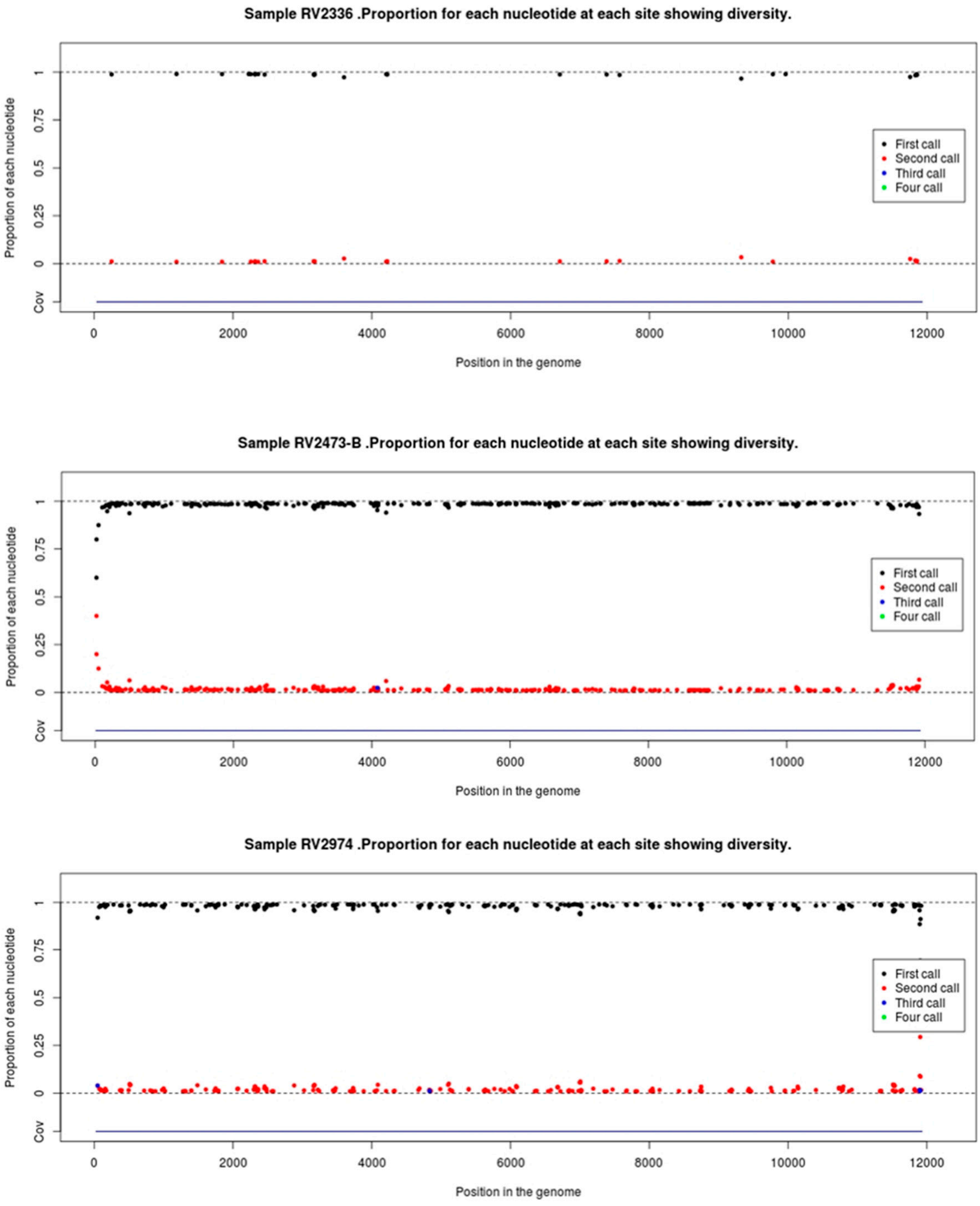

Figure S1. Positions at which heterogeneity is seen within the three EBLV-2 isolates from Stokesay castle. A. 2007 (RV2336), B. 2008 (RV2473), C. 2014 (RV2974). Mapping quality 30, call quality 30, error 0.01.
